# Quadruple common trunk of the axillary artery and high brachial division: a cadaveric study

**DOI:** 10.1007/s00276-026-03971-2

**Published:** 2026-08-12

**Authors:** Debyani Kishore, Purnima Adhikari, Rohini Punja

**Affiliations:** Department of Anatomy, Kasturba Medical College, Manipal Academy of Higher Education, Manipal, India

**Keywords:** Axillary artery, Brachial artery, Arterial variation, Cadaveric dissection, High division, Upper limb anatomy, Morphometry

## Abstract

**Purpose:**

This study describes three upper limb arterial variants identified incidentally during systematic cadaveric dissection, with characterisation of their morphometric features, embryological basis, and potential surgical relevance.

**Methods:**

Twenty formalin-fixed adult cadavers (40 upper limbs) were systematically dissected bilaterally. Vessel diameters, trunk lengths, and bifurcation levels were measured using digital Vernier callipers (accuracy ± 0.02 mm). Each variant was documented by photography and schematic diagram.

**Results:**

Three variants were identified (3/40 limbs, 7.5%). Case 1: a quadruple common trunk arising from the third part of the axillary artery, 25.3 mm distal to the lateral border of pectoralis minor, giving origin to the lateral thoracic (2.1 mm), subscapular (3.8 mm), circumflex scapular (2.6 mm), and posterior circumflex humeral arteries (1.9 mm). Case 2: high brachial division 15.5 cm proximal to the radial neck, with radial artery traversal of the bicipital aponeurosis. Case 3: high brachial division with a focal ulnar narrowing commencing 22.0 mm from the bifurcation, extending over 8.5 mm, with a minimum diameter of 1.4 mm resolving to 3.2 mm distally.

**Conclusion:**

Three anatomical variants are described with complete morphometric documentation. No identical configuration to Case 1 was identified in the reviewed literature. The findings contribute descriptive cadaveric data. Given the small series and cadaveric setting, all interpretations are offered with due caution.

## Introduction

The arterial system of the upper limb develops during embryonic weeks 7–9 through selective haemodynamic regression of a capillary plexus derived from the seventh cervical intersegmental artery [13, 14]. Channels with dominant blood flow persist and consolidate into the definitive named branches; channels with subordinate flow regress [13, 14]. This process determines the origin, calibre, and course of every named axillary and brachial branch, and disruption at any stage can produce persistent channels, aberrant origins, or premature bifurcations [13].

Variations in axillary artery branching are well documented across large cadaveric series. Common trunks incorporating two or three named branches have been reported from all three parts of the axillary artery [1, 2, 4, 8, 10, 17, 18, 21]. High division of the brachial artery—defined as bifurcation proximal to the interepicondylar line—has been documented with prevalences ranging from 1.5% to 25.0% in different series [6, 7, 11]. Both variant types have been noted in the literature as anatomical findings potentially relevant to axillary dissection, radial artery harvest, and antecubital vascular access, though the evidence underlying these associations derives primarily from cadaveric and case-level observation [9].

In the present study, three arterial variants were identified incidentally during systematic bilateral dissection of 20 cadavers. This report aims to describe and fully document each variant morphometrically and to note potential surgical relevance with reference to published literature. No claim of systematic prevalence study is made; confidence intervals are provided as descriptive context only.

## Materials and methods

### Cadaveric specimens and ethics

Twenty formalin-fixed adult cadavers (40 upper limbs) were dissected at the Department of Anatomy, Kasturba Medical College, Manipal, India. Cadavers were obtained through the institutional body donation programme. South Indian origin was inferred from institutional records; formal documentation of regional origin by an independent method was not undertaken and is acknowledged as a limitation. The study was approved by the Institutional Ethics Committee [IEC 66/2023] in accordance with the Declaration of Helsinki. Cadavers with documented upper limb trauma, prior vascular surgery, or gross deformity were excluded. Bilateral dissection was performed in every cadaver (40 limbs total). Twelve cadavers were male and eight were female, with ages ranging from 45 to 78 years (mean 61.3 years).

### Dissection

Skin, subcutaneous tissue, and deep fascia were removed by systematic layer dissection from the clavipectoral triangle to the cubital fossa. Pectoralis minor was fully exposed and its medial and lateral borders identified and marked before retraction, to permit accurate assignment of axillary artery branches to their correct part. Each named branch of the axillary artery was traced from its origin to its first division. The brachial neurovascular bundle was traced from the axilla to the neck of the radius. The bicipital aponeurosis was preserved intact throughout. All dissections were performed by qualified anatomists.

### Measurements

External diameters and trunk lengths were measured using digital Vernier callipers (+/-0.02 mm; Mitutoyo Corporation, Japan). Parameters recorded were: (i) distance from the lateral border of pectoralis minor to the common trunk origin; (ii) common trunk length and external diameter; (iii) external diameters of all named branches at their origins from the common trunk; (iv) level of brachial bifurcation as linear distance from the neck of the radius; and (v) for the focal ulnar narrowing in Case 3: distance from the bifurcation to the commencement of narrowing, length of the narrowed segment, minimum external diameter at the narrowing, and external diameter of the normal distal ulnar artery. All measurements were recorded by two independent observers; means are reported.

### Statistical analysis

This study is a case series of incidentally identified variants. Simple frequencies are reported as the primary measure. Confidence intervals calculated by the Wilson score method [23] are provided solely as descriptive context and should not be interpreted as indicating statistical robustness; no inferential statistics were applied.

## Results

Three arterial variants were identified in 3 of 40 limbs (7.5%) across three separate cadavers; the remaining 37 limbs (92.5%) showed anatomy conforming to standard descriptions [20]. All three variants were unilateral; no contralateral mirror variation was observed.

### Case 1: quadruple common trunk from the third part of the axillary artery

**Fig. 1:**
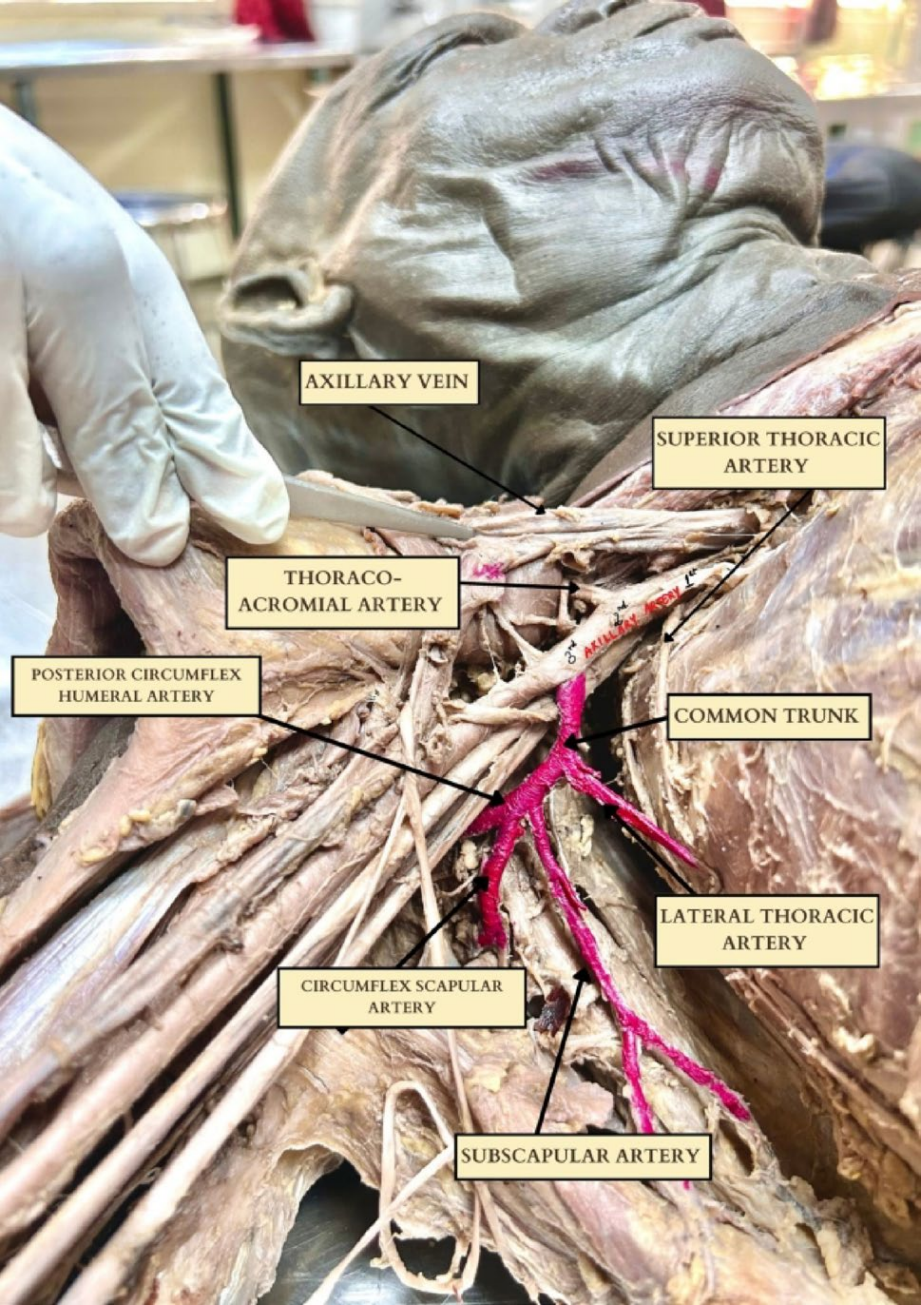
Cadaveric dissection of the right axillary region, Case 1. The common trunk (CT) arises from the third part of the axillary artery (3rd AA), 25.3 mm distal to the lateral border of pectoralis minor. The lateral thoracic artery (LTA), subscapular artery (SA), circumflex scapular artery (CSA), and posterior circumflex humeral artery (PCHA) all arise from the CT. The thoracoacromial artery (TA), superior thoracic artery (ST), and axillary vein (AV) are identified in their standard positions

**Fig. 2:**
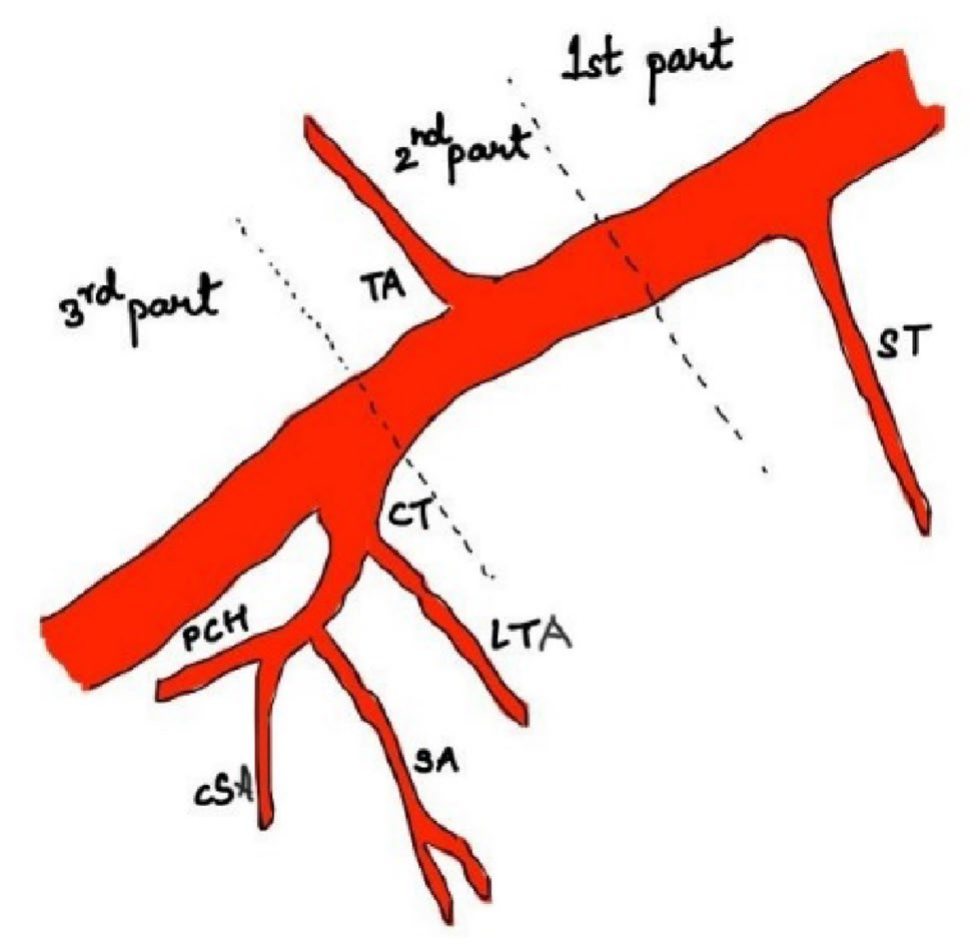
Schematic diagram of Case 1. ST superior thoracic artery; TA thoracoacromial artery; CT common trunk; LTA lateral thoracic artery; SA subscapular artery; CSA circumflex scapular artery; PCH posterior circumflex humeral artery. Dashed lines indicate the three parts of the axillary artery. The CSA arises directly from the CT as an independent branch; the SA also arises from the CT and divides subsequently into the thoracodorsal artery and muscular branches

In the right upper limb of a 65-year-old male, a common trunk was identified arising from the third part of the axillary artery (Figs. 1, 2). Both the medial and lateral borders of pectoralis minor were identified and marked during dissection. The common trunk origin was located 25.3 mm distal to the lateral border of pectoralis minor, confirming its position within the third part of the axillary artery. The trunk measured 12.5 mm in length and 8.2 mm in external diameter at its origin.

Four named vessels arose from the common trunk: (i) the lateral thoracic artery (LTA), 2.1 mm diameter, which passed medially to supply the lateral thoracic wall—its origin from the third-part trunk represents an ectopic position relative to its standard second-part or thoracoacromial origin [9]; (ii) the subscapular artery (SA), 3.8 mm diameter, after giving out a muscular branch seen supplying the subscapularis muscle subsequently continued as the thoracodorsal artery, which was confirmed by its supply to latissimus dorsi muscle; (iii) the circumflex scapular artery (CSA), 2.6 mm diameter, which arose as a separate, direct branch from the common trunk independent of the subscapular artery and passed through the triangular space; and (iv) the posterior circumflex humeral artery (PCHA), 1.9 mm diameter, which accompanied the axillary nerve through the quadrangular space. The superior thoracic, thoracoacromial, and anterior circumflex humeral arteries arose independently from their standard positions. The contralateral axillary artery was normal.

The branching configuration is clarified as follows: the CSA arose directly from the common trunk as an independent branch, while the SA also arose from the common trunk and subsequently continued as the thoracodorsal artery after giving out muscular branch to the subscapularis muscle. The thoracodorsal artery is the terminal continuation of the subscapular artery. Both the CSA and the SA were therefore distinct branches of the common trunk; the CSA did not arise from the SA in this specimen. This arrangement is illustrated schematically in Fig. 2. A review of the published literature identified no previously described quadruple common trunk from the third part of the axillary artery incorporating ectopic LTA with independent circumflex scapular artery origin (Table 1).

**Table 1:** Published axillary artery common trunk variants

Authors (Year)	Side	Part of axillary artery	Vessels in common trunk	No. of vessels	LTA involved
De Garis and Swartley [4]	Multiple	2nd/3rd	Various combinations; subscapular and circumflex humeral	2–3	Variable
Akhtar et al. [1]	R	2nd	LTA + subscapular artery	2	Yes
Astik and Dave [2]	Multiple	2nd/3rd	Subscapular + LTA; subscapular + ACHA + PCHA	2–3	Variable
Saeed et al. [14]	Bilateral	3rd	Subscapular + circumflex humeral arteries	2	No
Sarkar et al. [15]	Bilateral	Not specified	Muscular branch + LTA + subscapular + PCHA	4	Yes
Verma et al. [18]	L	2nd	Thoracoacromial + subscapular + ACHA + PCHA + accessory subscapular	5+	No
**Present study**	**R**	**3rd**	**LTA + SA + CSA + PCHA -- all four from a single third-part trunk**	**4**	**Yes (ectopic)**

*ACHA* anterior circumflex humeral artery; *PCHA* posterior circumflex humeral artery; *LTA* lateral thoracic artery; *SA* subscapular artery; *CSA* circumflex scapular artery. Bold row = present case

### Case 2: high division of the brachial artery with radial artery traversal of the bicipital aponeurosis

**Fig. 3:**
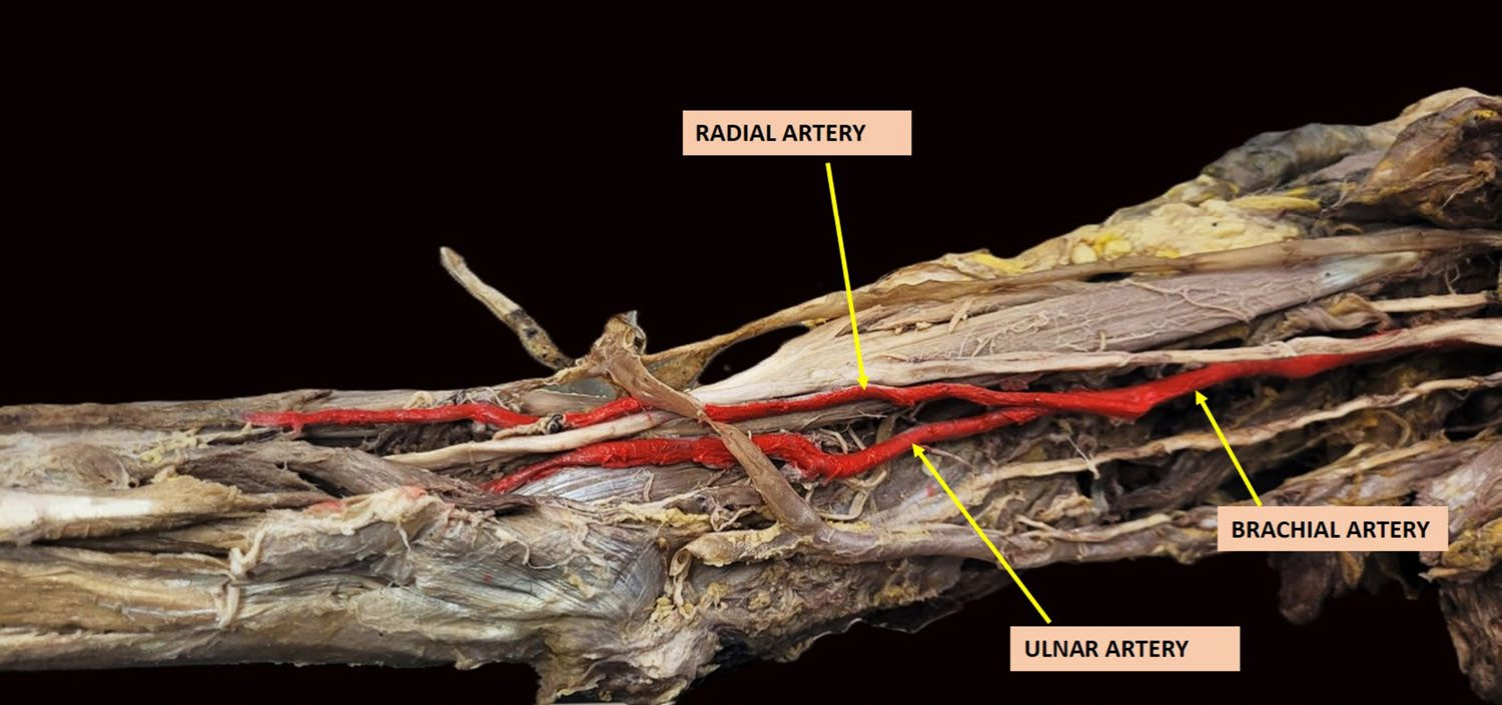
Cadaveric dissection of the right antecubital region, Case 2, vessels highlighted in red dye (primary figure). The brachial artery (BA), radial artery (RA), and dominant ulnar artery (UA) are labelled, demonstrating the high bifurcation level and relative calibres of the terminal branches

**Fig. 4:**
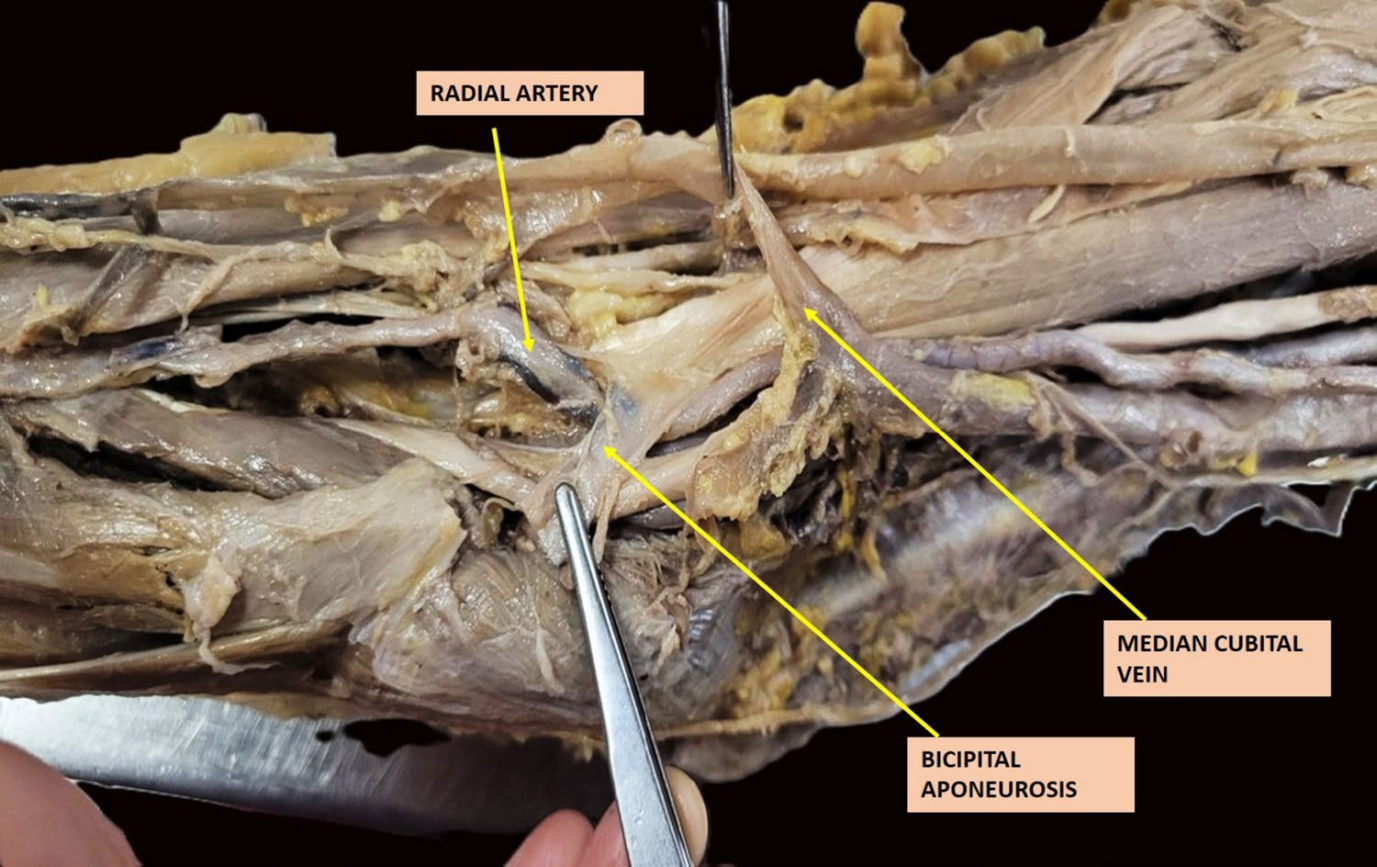
Same specimen as Fig. 3 before vessel highlighting. The radial artery (RA) is shown passing superficially through the bicipital aponeurosis (BA). The median cubital vein (MCV) is also labelled

In the right upper limb of a 65-year-old female, the brachial artery divided 15.5 cm (155 mm) proximal to the neck of the radius, above the interepicondylar line (Figs. 3, 4), satisfying the criterion for high division [11]. The radial artery—the smaller of the two terminal branches—passed superficially through rather than deep to the bicipital aponeurosis before continuing into the forearm. The ulnar artery, the dominant branch, maintained a course deep to the aponeurosis. The median nerve and other structures maintained their standard positional relationships. The contralateral limb was normal.

### Case 3: high division of the brachial artery with focal proximal ulnar narrowing

**Fig. 5:**
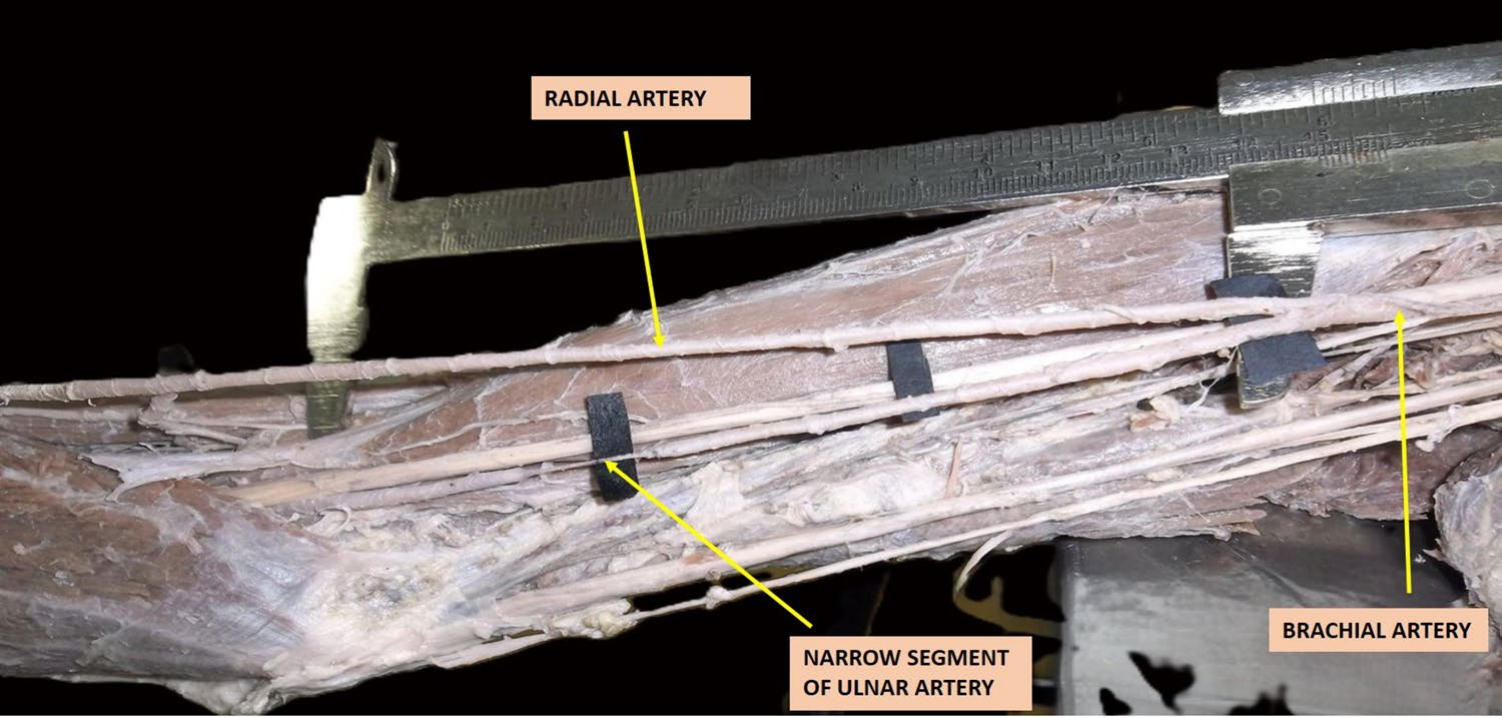
Cadaveric dissection of the right arm, Case 3. A Vernier callipers confirms the level of brachial bifurcation. The radial artery (RA), the narrow segment of the ulnar artery (UA*), and the brachial artery (BA) are labelled. The narrowed segment commences 22.0 mm from the bifurcation and extends over 8.5 mm

**Fig. 6:**
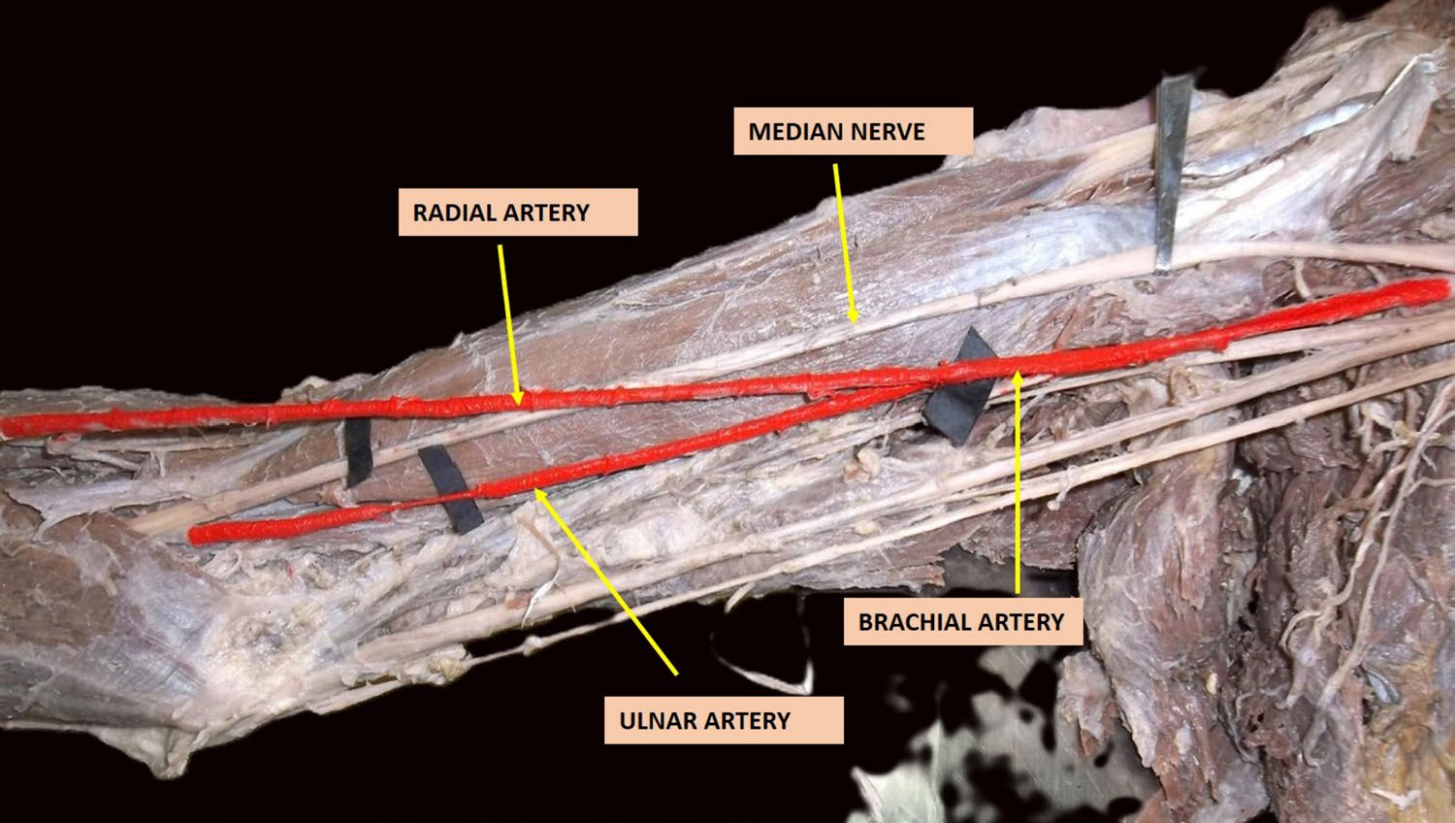
Same specimen as Fig. 5 with arteries highlighted in red dye. The radial artery (RA), ulnar artery (UA), brachial artery (BA), and median nerve (MN) are labelled

**Fig. 7:**
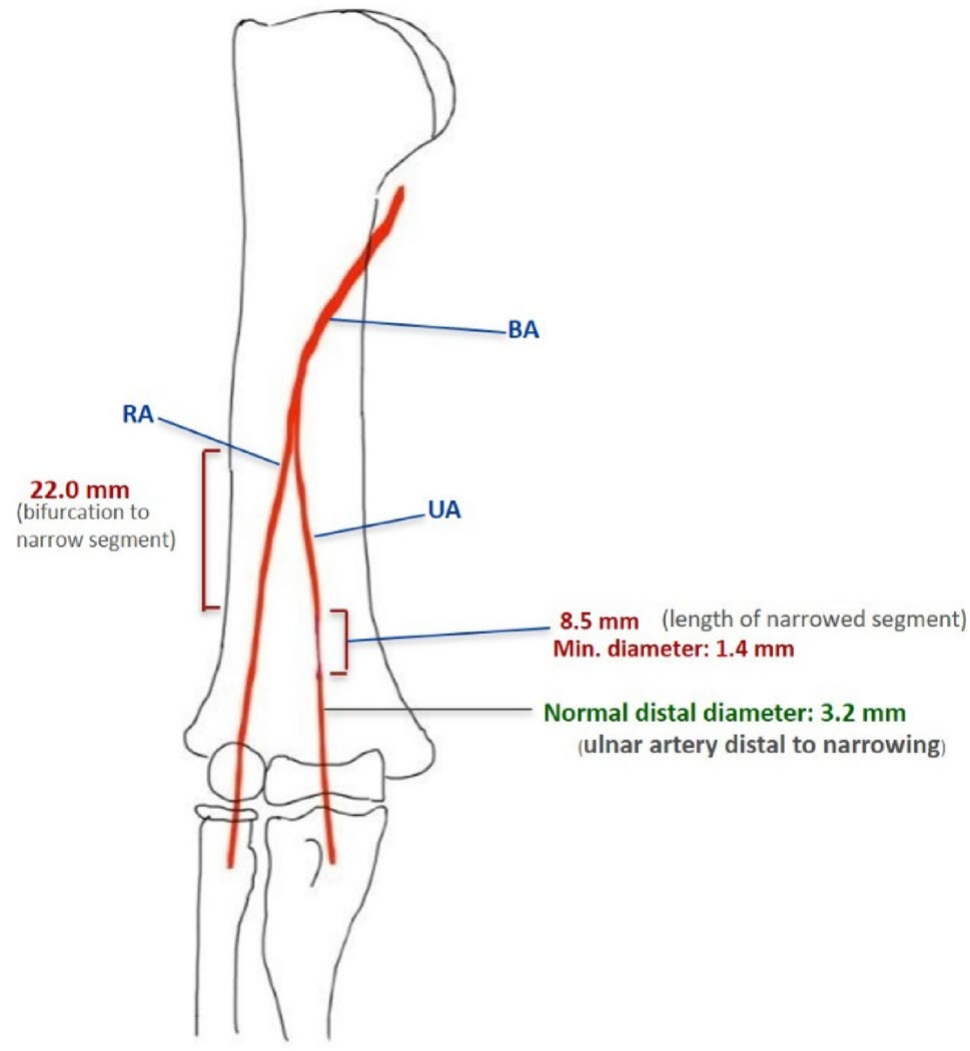
Schematic diagram of Case 3 illustrating high brachial division and the focal ulnar narrowing. The narrowed segment (hatched) commences 22.0 mm distal to the bifurcation, extends 8.5 mm, with minimum diameter 1.4 mm resolving to 3.2 mm distally. The radial artery (RA), ulnar artery (UA), and brachial artery (BA), are labelled

In the right upper limb of a 62-year-old male, the brachial artery bifurcated at the junction of the upper one-third and lower two-thirds of biceps brachii (Figs. 5, 6, 7). A focal segment of reduced external calibre was identified in the ulnar artery beginning 22.0 mm distal to the bifurcation and extending over a length of 8.5 mm. The minimum external diameter at the narrowing was 1.4 mm, resolving to a normal calibre of 3.2 mm distal to the narrowed segment. At dissection the vessel wall was patent and supple, with no extrinsic compression, luminal thrombus, intimal irregularity, or atherosclerotic change identified. The distal ulnar artery was normal in calibre and course. The contralateral limb was normal.

## Discussion

### The quadruple axillary common trunk: anatomical clarification and literature context

The classification of the common trunk in Case 1 as arising from the third part of the axillary artery is supported by direct morphometric measurement: the trunk origin was located 25.3 mm distal to the lateral border of pectoralis minor, which is the anatomical landmark defining the commencement of the third part [20]. Both borders of pectoralis minor were identified and marked during dissection before measurement was taken. The concern that the width of pectoralis minor might place the origin within the second part is acknowledged; however, the measurement was specifically taken from the lateral border, establishing the trunk position distal to that boundary and therefore within the third part.

Regarding vessel nomenclature in Case 1: the CSA arose as a direct, independent branch from the common trunk. The SA also arose from the common trunk and subsequently continued as the thoracodorsal artery after giving muscular branch to subscapularis muscle. This is in line with the standard definition of the subscapular artery as the parent vessel of the thoracodorsal artery [20]. The thoracodorsal artery is the terminal continuation of the subscapular artery. The CSA and SA were therefore distinct branches of the common trunk; the CSA originated ectopically from the trunk directly, rather than as a branch of the SA as is anatomically standard. This arrangement is accurately described and illustrated in Fig. 1b with standardised abbreviations.

Review of the literature on axillary artery branching patterns identified no previously described configuration combining a quadruple common trunk from the third part with independent CSA origin and ectopic LTA inclusion. Relevant published series are compared in Table 1. De Garis and Swartley [4] provided the earliest and largest systematic account of axillary artery variation; McCormack et al. [10] studied 750 extremities; Huelke [8] examined transverse cervical and dorsal scapular arterial patterns. Astik and Dave [2] described multiple branching variants in 40 cadavers. Sarkar et al. [18] described a quadruple arrangement from an unspecified part without LTA ectopia. Verma et al. [21] reported a five-vessel trunk from the second part. None involves the specific combination observed in Case 1. We state consistently throughout that no identical configuration was identified in the reviewed literature rather than claiming this variant is definitively the first ever reported. Comparable single-case cadaveric reports of previously undescribed arterial configurations continue to emerge in the upper limb literature [5, 16], reinforcing the value of systematic dissection documentation.

From a surgical perspective, recognition of a large common trunk in the third part of the axillary artery carrying multiple named branches is relevant to axillary lymph node dissection and flap surgery, as documented in the broader literature [9]. This is a general caveat grounded in published evidence and does not require specific inference from a single case.

### High brachial division

Both Cases 2 and 3 showed brachial bifurcation proximal to the interepicondylar line. High division is well documented; the systematic review by Hassall et al. [7] reported prevalences from 1.5 to 25.0% across series. The 5.0% rate in the present series is within this range. In Case 2, superficial radial artery traversal of the bicipital aponeurosis in association with high division has been reported previously [3, 7, 12] and is discussed in the literature in relation to radial artery harvest [9]. In Case 3, the focal ulnar narrowing, documented with objective morphometry demonstrated minimum diameter of 1.4 mm at the narrowing, resolving to 3.2 mm distally, was assessed macroscopically as a patent vessel without pathological features. Analogous calibre transitions in association with brachial high division have been reported by Vollala et al. [22]; the present case adds objective morphometric data to this category of observation.

### Embryological note

Embryological explanations for the variants observed are consistent with the published framework of axial plexus regression described by Rodriguez-Niedenführ et al. [13–15] and the intersegmental origin model of Yang et al. [24]. These interpretations are plausible inferences from comparative developmental anatomy; they are not conclusions that can be directly verified from cadaveric morphology alone. Singer’s haemodynamic retention hypothesis [19] provides a mechanistic framework for common trunk persistence; its application to the present case is theoretical. All embryological statements in this manuscript are framed as explanatory possibilities rather than developmental conclusions.

### Limitations

This study is a case series of three incidentally identified variants from a single institution. The small total dissection series (20 cadavers) does not permit reliable prevalence estimation; frequency figures are reported for completeness but should not be interpreted epidemiologically. The determination of geographic origin was based on institutional records without formal independent verification. Haemodynamic significance of the variants cannot be assessed from cadaveric morphology. The embryological interpretations available in the literature are noted for context; none is applied as a conclusion from the present observations.

## Conclusion

Three upper limb arterial variants were identified and fully documented during systematic cadaveric dissection: a quadruple common trunk from the third part of the axillary artery with independent circumflex scapular artery origin and ectopic lateral thoracic artery; high brachial division with superficial radial artery traversal of the bicipital aponeurosis; and high brachial division with a morphometrically documented focal ulnar narrowing (commencing 22.0 mm from the bifurcation, extending 8.5 mm, minimum diameter 1.4 mm resolving to 3.2 mm distally). No identical configuration to Case 1 was identified in the reviewed literature. The findings are descriptive cadaveric observations based on a small series; they are reported with complete morphometric documentation for the benefit of anatomists and surgeons encountering similar findings.

## Data Availability

No datasets were generated or analysed during the current study.
